# Preference of blood pressure measurement methods by primary care doctors in Hong Kong: a cross-sectional survey

**DOI:** 10.1186/s12875-020-01153-6

**Published:** 2020-05-26

**Authors:** Eric Kam Pui Lee, Ryan Chun Ming Choi, Licheng Liu, Tiffany Gao, Benjamin Hon Kei Yip, Samuel Yeung Shan Wong

**Affiliations:** 1 Jockey Club School of Public Health and Primary Care, The Chinese University of Hong Kong, Hong Kong SAR, China; 2 Room 402, 4/F, Jockey Club School of Public Health and Primary Care building, Prince of Wales Hospital, Shatin, NT, Hong Kong SAR, China; 3 Faculty of Medicine, The Chinese University of Hong Kong, Hong Kong SAR, China

**Keywords:** Ambulatory BP monitoring, Home BP monitoring, Hypertension, Automated office BP, Blood pressure measurement, Doctors’ behaviour, Primary care

## Abstract

**Background:**

Hypertension is the most common chronic disease and is the leading cause of morbidity and mortality. Its screening, diagnosis, and management depend heavily on accurate blood pressure (BP) measurement. It is recommended that the diagnosis of hypertension should be confirmed or corroborated by out-of-office BP values, measured using ambulatory BP monitoring (ABPM) and home BP monitoring (HBPM). When office BP is used, automated office BP (AOBP) measurement method, which automatically provides an average of 3–5 BP readings, should be preferred. This study aimed to describe the BP measurement methods commonly used by doctors in primary care in Hong Kong, to screen, diagnose, and manage hypertensive patients.

**Methods:**

In this cross-sectional survey, all doctors registered in the Hong Kong “Primary Care Directory” were mailed a questionnaire, asking their preferred BP-measuring methods to screen, diagnose, and manage hypertensive patients. Furthermore, we also elicited information on the usual number of office BP or HBPM readings obtained, to diagnose or manage hypertension.

**Results:**

Of the 1738 doctors included from the directory, 445 responded. Manual measurement using a mercury or aneroid device was found to be the commonest method to screen (63.1%), diagnose (56.4%), and manage (72.4%) hypertension. There was a significant underutilisation of ABPM, with only 1.6% doctors using this method to diagnose hypertension. HBPM was used by 22.2% and 56.8% of the respondents to diagnose and manage hypertension, respectively. A quarter (26.7%) of the respondents reported using only one in-office BP reading, while around 40% participants reported using ≥12 HBPM readings. Doctors with specialist qualification in family medicine were more likely to use AOBP in clinics and to obtain the recommended number of office BP readings for diagnosis and management of hypertension.

**Conclusion:**

Primary Care doctors in Hong Kong prefer to use manual office BP values, measured using mercury or aneroid devices, to screen, diagnose, and manage hypertension, highlighting a marked underutilisation of AOBP and out-of-office BP measuring techniques, especially that of ABPM. Further studies are indicated to understand the underlying reasons and to minimise the gap between real-life clinical practice and those recommended, based on scientific advances.

**Trial registration:**

Clinicaltrial.gov; ref. no.: NCT03926897.

## Background

Hypertension (HT) is the most common chronic disease globally, affecting around one-third of the world’s adult population [1]. It can lead to numerous debilitating cardiovascular complications, and HT-related cardiovascular diseases remain the leading cause of death in China and worldwide [2]. As HT is mostly asymptomatic, its detection, diagnosis, and treatment relies heavily and exclusively on accurate blood pressure (BP) measurements [3].

Traditionally, HT was diagnosed solely by repeated measurements of BP in the doctor’s office (office BP). However, office BP measurements often wrongly categorise patients, due to a significant proportion having white-coat (~ 30–40%) and masked (~ 10%) hypertension in clinical practice [4]. Out-of-office BP measurements, including ambulatory blood pressure monitoring (ABPM) and home blood pressure monitoring (HBPM), have therefore taken up an indispensable role in the diagnosis of HT. In fact, mean BP values obtained by ABPM and HBPM, are better predictors of future cardiovascular events and risk of fatality than those obtained during routine follow-ups in the clinic [5–7]. Furthermore, studies have found ABPM to be the most cost-effective method of diagnosing HT, due to its specificity in identifying truly hypertensive patients who need appropriate management [8, 9]. These findings have led to changes in recommendations made by international guidelines. Out-of-office BP is now considered the preferred method to diagnose HT according to the National Institute for Health and Clinical Excellence (NICE) guidelines, the American College of Cardiology/American Heart Association Task Force guidelines, and the Hypertension Canada guidelines [3, 10, 11]. Out-of-office BP measurement is also accepted as an alternative to in-office BP assessment for diagnosis of HT as per the European Society of Hypertension and European Society of Cardiology guidelines [12].

Regardless, office BP measurement still plays a major role in screening and managing HT. Not only is this method feasible, but also, most existing studies on the topic have used office BP measurements as primary end-points [11]. Until more studies utilise BP readings obtained through ABPM or HBPM as outcome measures, office BP measurement will remain the primary method of estimating BP values for determining the need for, and titrating anti-hypertensive treatment [11]. While using office BP, all existing HT guidelines suggest strict adherence to BP measurement techniques because a standardised protocol is important for accurate BP estimation [3, 10–12]. However, healthcare professionals have shown poor adherence to these recommendations, which results in inaccurate measurements [13]. Considering these manual errors, a new office BP measurement method called “automated office BP” (AOBP), which involves automatic, electronic measurement of 3–5 BP values to produce a mean value, was developed [14]. AOBP values, as compared to those obtained via traditional office BP-measuring methods (oscillometric or mercury sphygmomanometers that are manually used by patients or healthcare professionals), were recently found to provide BP estimations that were closer to those obtained via ABPM, which is generally considered the “gold standard” of clinical BP measurement [14]. Therefore, AOBP assessment is recommended by several international HT societies [10, 12]. When office BP is measured, use of automatic BP or semi-automatic machines is generally preferred over manual sphygmomanometers [12]. Besides BP measurement techniques, the number of BP values used to diagnose and guide management of HT is important. While using office BP, for instance, international HT guidelines suggest taking multiple readings at each clinic visit because a single BP value is not reliable [15]. Similarly, while using HBPM, increasing amount of evidence suggests that at least 12–14 BP readings should be taken, both in mornings and evenings over 1-week, to allow accurate diagnosis and management of HT; currently, the Hong Kong guideline suggests the least of 24 BP readings [16, 17].

Although, it is known that clinicians show poor compliance to BP measurement techniques in the office, very few studies have evaluated the exact technique routinely used by doctors to measure BP in the clinical setting. A recent survey of family physicians in Canada found that despite the emphasis placed by Canadian HT guidelines on the use of ABPM and AOBP, only 14.4% participants used ABPM for diagnosing HT [18]. Other similar studies had small sample-sizes, or were older [19–21]. Furthermore, no such study has been conducted in the Chinese population. Knowing how exactly doctors measure BP can help determine the gap between scientific recommendations and routine clinical practice and further help us identify the need for training and proper framing of guidelines, such that accurate BP measuring techniques are widely implemented in the clinical setting.

In this survey study including primary care doctors, we hypothesised that: (i) a large proportion of doctors used ABPM or HBPM for diagnosis of HT, (ii) AOBP was the preferred method for office BP measurement, screening, diagnosis, and management of HT, and (iii) when manual/traditional office BP and/or home BP measurement methods were used, the recommended number of BP readings (at least duplicate readings for office BP and ≥ 12 readings for home BP) were obtained.

## Methods

### Study subjects

This was a cross-sectional survey conducted from May to July in 2019. In Hong Kong (HK), the department of Health has built an online platform called the “Primary Care Directory”, such that the general public can conveniently locate primary care doctors. Registered doctors who consider themselves as primary medical caregivers, can enrol themselves in this online directory on a voluntary basis. These doctors include general practitioners (who do not receive further training after graduation from their medical schools), family medicine specialists in training, trained family medicine specialists, and doctors of other specialities. All doctors enrolled in the “Primary Care Directory” during the study period, were invited by mail to participate in the study. All doctors were sent the questionnaire by mail thrice, at 2-week intervals, to enhance the response rate. Each questionnaire was numbered to avoid a duplicate response. An information sheet was attached to the questionnaire, explaining the study details.

The study was approved by the CUHK Survey and Behavioural Research Ethics Committee (ref no: *SBRE-18-563*.) and was registered in the clinical trial registry (clinicaltrial.gov; ref. no.: *NCT03926897*).

### Instrument

We framed the survey questionnaire using questions from the National Survey of Canadian Family Physicians, to ensure that the results obtained were comparable [18]. The questions were developed by experts in family medicine, survey methodology, and BP measurement (see appendix).

The questions included: “What is your routine method used to measure BP in patients being screened for HT?” (Q1 - for screening); “Once a routine screening BP suggests that HT may be present, what is your usual method for measuring BP to make a diagnosis of HT?” (Q2 - for diagnosis); and “What are your routine methods used to measure BP in patients taking antihypertensive treatment (lifestyle or medications)?” (Q3 - for management).

The possible answers included: “Manual BP in the office with mercury or aneroid device”; “Manual BP in the office with an electronic device”; “Automated office BP machines (AOBP) including BpTRU, Omron907XL, or Microlife WatchBP Office”; “Other patient-activated electronic devices in the office”; “Electronic BP kiosks”; “Ambulatory BP monitoring”; “Home BP monitoring”; and “Others”. The alternatives ABPM and HBPM were omitted from the answers to the screening question (Q1) because, these diagnostic modalities were not considered feasible for screening of HT. Conversely, multiple answers were allowed for the monitoring and management question (Q3) because it was possible for the doctors to use > 1 type of BP measurement method to guide treatment (Table 2). These aforementioned questions were the same as in the original Canadian National survey.

In addition, we also asked: (i) the total number of HBPM readings used to diagnose or manage HT (if the doctor selected HBPM as their preferred technique in response to previous questions) and (ii) the number of office BP readings obtained per clinic visit (if the doctor selected office BP measurement as their preferred technique in previous questions). Demographic data including age, sex, work-sector, and specialist status were collated.

### Sample size

Our primary outcome measure was the proportion of doctors using various BP measurement methods (e.g. the proportion of doctors using out-of-office BP to diagnose HT). However, we had insufficient data to determine the sample size required, due to lack of previous research in the Chinese population. According to the National Survey of Canadian Family Physicians, the proportion of doctors using various BP measurement methods ranged from 14.4–54.2% [18]. Presuming a margin of error of ~ 5%, using 95% confidence interval (CI), considering the total number of doctors in the Primary Care Directory (*n* = 1951), and assuming the expected proportion of 50% (which would require the largest number of participants), we determined the size of the required study population to be 321 participants.

### Statistical analysis

The statistical analysis was conducted using the SPSS 24 (IBM Corp. Released 2016. IBM SPSS Statistics for Windows, Version 24.0. Armonk, NY: IBM Corp) software. The primary outcomes were proportions of BP measurement methods that doctors in HK used, to screen, diagnose, and manage HT. These were presented as percentages. The relationship between each BP measurement method used and applicable demographic data were first analysed using the Chi-Square test or the Fisher-exact test. Multiple logistic regression was used when > 1 statistically significant predictors defined by a *p*-value of ≤0.05, were found in the aforementioned univariate analysis. We obtained odds ratios and 95% CIs for each independent variable. A two-sided p-value of ≤0.05 was considered to be statistically significant. Missing data were excluded, and no imputation was conducted.

## Results

Of the 1951 doctors in the directory, 213 were excluded due to various reasons including incorrect contact information (*n* = 90) or emigration, not currently practising, retirement or death of the doctor (*n* = 123). Totally, 445 doctors responded to the survey, with an overall response rate of 25.6% (445/1738).

Demographic data of participants has been summarised in Table 1. Most participants were > 60 years of age (53.7%), male (81.9%), working in the private sector (95.9%) and were not specialists in family medicine (78.9%).

**Table 1:** Demographic data of respondents

Characteristic	Number (%)
Age (years)	
• < 35	5 (1.1%)
• 35–39	11 (2.5%)
• 40–44	35 (8.0%)
• 45–49	36 (8.3%)
• 50–54	59 (13.5%)
• 55–59	56 (12.8%)
• 60 or above	234 (53.7%)
Sex	
• Male	354 (81.9%)
Work sector	
• Hospital Authority	7 (1.6%)
• Private Clinic or Hospital	418 (95.9%)
• University Clinics	2 (0.5%)
• Academics	1 (0.2%)
• Others	8 (1.8%)
Specialist degree in family medicine	
• Yes	92 (21.1%)

### BP screening

The most commonly used screening method was manual BP measurement using a mercury or aneroid device (63.1%), followed by manual measurement with an electronic device (20.2%). Only 13.7% (*n* = 59) participants reported using AOBP for screening of HT (Table 2).

**Table 2:** Practices of family doctors to screen, diagnose, and manage hypertension

BP Measurement Method	Screening	Diagnosis	Management (multiple choices allowed)
Manual BP in the office with mercury or aneroid device	272 (63.1%)	216 (56.4%)	320 (72.4%)
Manual BP in the office with an electronic device	87 (20.2%)	48 (12.5%)	128 (29%)
AOBP measured using BpTRU, Omron 907XL, or Microlife WatchBP Office (Welch Allyn ProBP 2400)	59 (13.7%)	20 (5.2%)	88 (19.9%)
Other patient-activated electronic devices in the office	10 (2.3%)	7 (1.8%)	24 (5.4%)
Electronic BP kiosks	3 (0.7%)	1 (0.3%)	8 (1.8%)
Ambulatory BP monitoring (24-h to 48-h BP monitoring)	N/A	6 (1.6%)	18 (4.1%)
Home BP monitoring		85 (22.2%)	251 (56.8%)
Others	0 (0.0%)	0 (0.0)	3 (0.7%)
*Missing*	*14*	*62*	*3*

Abbreviations: *BP* blood pressure, *AOBP* automated office BP

In the univariate analysis, doctors were found to be more likely to screen for HT using AOBP as compared to other BP measurement methods if: (i) they were < 60 years of age (46.5% vs. 23.7%, *p* < 0.001), (ii) female (46.1% vs. 31.9%; *p* = 0.019), and (iii) specialists in family medicine (47.7% vs 30.7%; *p* = 0.003). On further logistic regression analysis of these factors, AOBP was found to be preferred by doctors who were family medicine specialists (OR 2.1; 95% CI: 1.3–3.5) or < 60-years of age (OR 2.7; 95% CI: 1.7–4.1) (Table 3). Female doctors did not continue to show significant correlation with AOBP use.

**Table 3:** Relationship between demographic data and BP measurement behaviour

	Using AOBP screening (vs. using other methods)	using out-of-office BP for HT diagnosis (vs. using in-office methods)	Using AOBP during HT treatment (vs. not using AOBP for monitoring)	Using HBPM for HT treatment (vs. not using HBPM for monitoring)	Taking multiple readings in single clinic visit	Taking ≥12 HBPM readings
age < 60	**OR 2.7 (95% CI: 1.7–4.1; p < 0.001)**	RR 0.95 (*p* = 0.78)	**OR 3.2 (95%CI: 1.9–5.4; p < 0.001)**	RR 1.06 (*p* = 0.45)	**OR 0.39 (95%CI:0.24–0.63;*p*** **< 0.001)**	RR 0.81 (*p* = 0.086)
sex = female	OR 1.4 (95%CI: 0.85–2.47; *p* = 0.173)	RR 1.37 (*p* = 0.16)	OR 1.44 (95%CI: 0.85–2.4; *p* = 0.18)	RR 1.04 (*p* = 0.71)	RR 0.93 (p = 0.45)	**RR 0.60 (p = 0.43)**
work = private	RR 1.18 (*p* = 0.66)	RR 1.69 (*p* = 0.39)	RR 0.58 (*p* = 0.14)	RR 1.15 (*p* = 0.52)	RR 0.92 (*p* = 0.63)	RR 1.38 (*p* = 0.57)
FM specialist	**OR 2.1 (95%CI: 1.3–3.5; p = 0.003)**	RR 1.20 (*p* = 0.41)	RR 1.35 (p = 0.16)	RR 0.95 (p = 0.63)	**OR 2.3 (95%CI:1.2–4.5;*p*** **= 0.017)**	RR 1.00 (*p* = 0.99)

Abbreviations: *BP* blood pressure, *AOBP* automated BP measurement, *HT* hypertension, *HBPM* home BP monitoring, *FM* Family Medicine

^a^*OR* Odd ratio, *RR* relative risk, *95%CI* 95% confidence intervals

^b^Odd ratio is presented if the predictors are analysed by multiple logistic regression (*see* statistical method)

### Diagnosis of HT

The most common method for diagnosing HT was manual measurement in the doctor’s office using a mercury or an aneroid device (56.4%), followed by HBPM (22.2%). Only 1.6% (*n* = 6) participants reported using ABPM for diagnosis of HT (Table 2). The use of out-of-office BP assessment techniques (HBPM or ABPM) to diagnose HT was not found to be associated with any demographic characteristics, including age, sex, work-sector (private versus non-private sector), and specialist status.

### HT management

The most commonly used method to monitor BP in patients with HT was manual measurement using a mercury or an aneroid device (72.4%). While one-fifth (19.9%) of study participants also reported using AOBP, more than half of the respondents (56.8%) used HBPM to guide medical treatment.

Univariate analysis showed that doctors < 60 years of age (29.4% vs 12.1%; *p* < 0.001) and female medical practitioners (28.2% vs 17.7%; *p* = 0.034) were more likely to use AOBP for monitoring and management of HT. In the logistic regression model, only doctors < 60 years of age were observed to be associated with the use of AOBP (OR 3.2; 95% CI: 1.9–5.4) to guide management of HT. The use of HBPM for monitoring was not associated with any of the demographic factors including age, sex, work-sector (private versus non-private sector), and specialist status.

### Number of BP readings taken in HBPM and office BP assessment

Totally, 26.7% of doctors relying on office BP measurements, reported taking only one BP reading during a clinic visit (*n* = 98). Doctors > 60 years of age (80.9% vs 62.8%; *p* < 0.001), and family medicine specialists (83.3% vs 70%; *p* = 0.024) were found to be more likely to take multiple BP readings (i.e. ≥2 readings). In the logistic regression analysis, both these predictors retained their significance. Doctors aged > 60 years (OR 2.6; 95% CI: 1.6–4.2), and specialist family medicine practitioners (OR 2.3; 95% CI: 1.2–4.5) were more likely to take > 1 BP reading during each clinic visit.

In answer to the question, “how many times do you ask patients to take BP on the day of monitoring?”, if HBPM was used, 32.9% (*n* = 83/252) doctors reported asking patients to only measure BP once on the day(s) of monitoring. Overall, there was large variation in the number of HBPM readings used to diagnose and monitor HT (range: 1–120). The mean number of HBPM readings used was 13.5, with a standard deviation of 15.5. Only 39.8% (*n* = 90/226) of the participants obtained ≥12 HBPM readings for diagnosis or management of HT. Female doctors were less likely to obtain adequate number of HBPM readings (≥12) (25.6% vs 42.4%; *p* = 0.43) (Table 3).

## Discussion

The current study found a great discrepancy between BP measuring methods recommended by international guidelines and those used in routine clinical practice by general practitioners in HK, who treat most people with hypertension. Majority of general practitioners used manual office BP-measuring methods, particularly using mercury or aneroid devices, for routine screening (63.1%), diagnosis (56.4%), and management (72.4%) of HT. Furthermore, while using office BP, many doctors (26.7%) reportedly obtained only one BP reading for diagnosis and/or treatment. Similarly, 60% doctors took < 12 HBPM readings for diagnosis or management of HT. On the other hand, ABPM was prominently underutilised, with only ~ 1% of doctors using this method to diagnose HT. The other out-of-office BP measurement method, HBPM, was used by less than one-fifth of doctors for diagnosis (22.2%), though around half of the respondents (56.8%) used HBPM to guide management of HT.

Despite manual office BP measurement being most commonly used by our respondents, it is the least accurate of all methods and therefore is least able to predict cardiovascular events. Further, it is prone to assessment errors due to factors such as inadequate rest period prior to BP measurement, carrying on a conversation during BP measurement, obtaining inadequate numbers of BP readings, rounding-errors when recording BP value, and excessively rapid manual deflation of the BP cuff [22].

The over-dependence on manual office BP and the underutilisation of out-of-office BP measurements mean that a substantial proportion of patients are over-treated or undertreated if they have white-coat hypertension or masked hypertension, respectively [4]. Researchers have shown that ≤30% patients could receive unnecessary treatment, if hypertension is solely diagnosed based on office BP values, as they could simply have white-coat hypertension [4]. A few studies have suggested the underlying reasons behind this preference for BP measurement methods. An Australian primary care study found that general practitioners were uncertain about the best way to measure BP, were unsure about the cut-off values for out-of-office BP estimation, did not have enough time to discuss techniques or results of ABPM or HBPM with the patients, and did not have the resources to prescribe or provide HBPM/ABPM [23]; these general practitioners suggested that a dedicated primary care guideline was needed [23]. Similarly, doctors are often concerned about whether the patient could master HBPM techniques and follow the strict measurement protocols required, to obtain accurate readings [24]. ABPM may also be perceived by doctors as inaccessible, expensive, inaccurate, and poorly tolerated [24]. Similar research in Chinese and Asian populations, especially in Hong Kong, is lacking. Although, several studies have described possible barriers to using out-of-office BP measurement methods, more studies are needed to understand how to implement accurate BP assessment techniques (e.g. ABPM/AOBP) in primary care. The current study also found that family medicine specialists were more likely to use AOBP in clinics and also to obtain enough office BP readings for diagnosis and management of HT, suggesting that targeted medical education can modify BP-measurement behaviour.

Only a few similar studies exist in the available literature. An online survey, which was conducted by The College of Family Physicians of Canada and involved 774 family physicians (response rate, 16.2%), found that AOBP was a common method to screen (42.9%), diagnose (31.1%), and manage (59.2%) HT, while ABPM was reported as the primary diagnostic tool for HT by 14.4% of the respondents [18]. A recent study in the United Kingdom (UK) surveying 489 patients, who self-reported to have BP measured during their last clinic visit, found that only one BP reading was obtained in 286 (59.6%) patients [25]. Considering the findings of the UK study, around 70% of doctors in our study obtained more than 1 clinic reading when manual BP measurement was used, which was higher than the UK study [25]. However, the authors of the UK study commented that their local guideline suggested duplicate office BP measurements only in patients with high BP readings, and this may explain the low duplicate office BP measurement rate [25]. Moreover, considering the findings of the Canadian study, significantly fewer doctors in HK used AOBP and ABPM for diagnosis and management of HT [18]. The reasons for the choice of BP measurement method cannot be delineated from the current study, and relevant studies on the topic are lacking in the Asian/Chinese population. However, we hypothesised that this may be due to a lack of relevant guidelines in HK. Although, the HK primary care guidelines have discussed the technique of HBPM at length, both ABPM and AOBP are not mentioned; the reasons behind omitting ABPM and AOBP were not discussed in the guideline [17]. In this study, despite underuse of out-of-office BP measurements to diagnose HT, more than half of the doctors (56.8%) incorporated HBPM readings to manage HT, suggesting the effect of the local guideline and feasibility of using HBPM in the local population. Secondly, most of our study participants were old and worked in the private sector. These doctors may have used the manual technique over many years and considered it a way to build rapport with patients and demonstrate their expertise [26]. Furthermore, patients may be used to, and will expect manual BP measurements, due to its widespread local use [26]. Finally, doctors in the private sector are able to conduct patient consultation for longer periods and can afford to spend time taking systematic manual measurements. These factors may at least partially explain the significant underutilisation of ABPM and AOBP in primary care in HK. A detailed qualitative study in HK, is needed to further ascertain our results and hypotheses.

Despite this being one of the first few studies to describe doctors’ preferences with respect to BP measurement methods, the study has certain limitations. Although, our response rate was low at 25.6%, this is comparable to, or even higher than those observed in previous similar surveys and e-mail studies involving doctors [18, 19]. The reason behind such low response rate could not be delineated by the current data but may have been due to reasons such as: (i) provision of incorrect mailing addresses, which meant that the questionnaire did not reach the doctors; (ii) the doctors were too busy to respond; (iii) doctors who did not usually treat patients with hypertension might have found the study irrelevant to them; and (iv) doctors who were unsure about their technique of measuring blood pressure correctly and might not have wanted to respond to the questionnaire. Furthermore, the primary care directory did not contain detailed demographic data and it was unclear if the demographics of our participants were similar to those of the non-respondents. Secondly, our results might have overestimated the number of doctors who used AOBP or out-of-office BP measurements because (i) the respondents were more likely to be interested and knowledgeable in BP measurements than non-respondents; and (ii) to ensure social desirability, respondents might have answered the questionnaire according to the international guidelines rather than describing techniques used in their actual clinical practices. However, these factors make our conclusion of underutilisation of out-of-office BP and AOBP measurements, even more robust. Thirdly, most of our respondents worked in the private sector and the applicability of our results to the doctors working in the public sector is unknown. Doctors working in the public sector might not have responded to the questionnaire because, they often do not have the freedom to choose a particular BP measurement method, which is determined by their respective departmental authority. The use of out-of-office BP measurement in the public sector is further limited by availability of resources and it is predicted that the use of out-of-office BP readings in these clinics is even less common. For example, the current public primary care department guideline only recommends ABPM (free of charge) in patients with suspected white-coat hypertension and the waiting time for these patients is currently more than 1 year. Similarly, patients seen in these government-funded clinics usually lack resources or higher education and thus may not be able to afford self-financed HBPM, although HBPM machines can be easily bought in HK. Furthermore, primary care doctors working in public sector have very short consultation time-intervals (often only 2–5 min/patient) and limited space (i.e. a quiet room where the patients could stay for 5 min for AOBP measurements), which are common barriers to AOBP and out-of-office BP measurements [27]. On the contrary, doctors working in the private sector can choose from a range of available BP measurement methods, have the time to counsel patients, and in turn, their patients might be more willing to bear the expense and effort required for ABPM/HBPM. In HK private sector, the cost of out-of-office BP measurements is often paid by patients and there is currently no reimbursement from the insurance companies or government. Lastly, we have only included doctors registered in the primary care directory and it is not known how representative this sample is to the whole population of primary care doctors in HK.

## Conclusion

In conclusion, we found that despite scientific advances and recommendations made by international guidelines, doctors administering primary care in HK still prefer to use manual office BP measurement, especially using mercury or aneroid devices, to screen, diagnose, and treat HT. There was a marked underutilisation of AOBP and out-of-office BP measuring techniques, especially that of ABPM. Studies are needed to understand the underlying reasons to minimise the gap between real-life clinical practice and use of recommended evidence-based methods. These could help us devise practical solutions so that accurate BP measurement methods are widely implemented in the primary care setting in HK.

## Data Availability

The datasets used and/or analysed during the current study are available from the corresponding author on reasonable request.

## Abbreviations

ABPM: Ambulatory BP monitoring
AOBP: Automated office BP
BP: Blood pressure
HBPM: Home BP monitoring
HK: Hong Kong
HT: Hypertension
